# The Impact of Moderate Hypophosphatemia on the Clinical Management of Primary Hyperparathyroidism

**DOI:** 10.7759/cureus.44115

**Published:** 2023-08-25

**Authors:** Elif Güneş, Mutlu Güneş

**Affiliations:** 1 Department of Endocrinology, Metabolism and Diabetes, Health Sciences University, Bursa State Hospital, Bursa, TUR; 2 Department of Endocrinology, Metabolism and Diabetes, Health Sciences University, Bursa Yuksek Ihtisas Training and Research Hospital, Bursa, TUR

**Keywords:** urinary calcium, hypercalcaemia, parathormone, primary hyperparathyroidism, hypophosphataemia

## Abstract

Background and objective

The impact of moderate hypophosphatemia (hypoP) on primary hyperparathyroidism (PHPT) and its use as an independent surgical criterion has not been adequately evaluated in the literature. In light of this, we conducted this study to address the scarcity of data on this topic.

Methods

We conducted a retrospective evaluation of data related to 164 (133 females and 31 males) patients with PHPT who met the criteria for inclusion in the study. HypoP, which is indicated by phosphorus (P) levels lower than 2.5 mg/dL, was found in 78 (47.5%) patients, and moderate hypoP (1-1.99 mg/dL) was found in 25 patients (15.2%).

Results

PHPT severity was worse in hypoP patients than non-hypoP patients, as evidenced by higher levels of mean serum calcium (12.9 ±1.0 mg/dL vs. 11.1 ±0.3 mg/dL respectively, p<0.001), parathormone (PTH) [median (interquartile range, IQR): 455.3 (455.3) ng/L vs. 124.0 (84.0) ng/L respectively, p<0.001] and mean 24-hour urinary calcium (414.6 ±168.5 mg/day vs. 291.5 ±161.4 mg/day respectively, p=0.026) as well as lower levels of mean BMI (25.6 ±3.9 kg/m^2^ vs. 29.0 ±4.0 kg/m^2 ^respectively, p=0.18) and mean 25-hydroxy vitamin D3 (13.8 ±7.3 µg/L vs. 18.2 ±7.8 µg/L respectively, p=0.001). Among the whole study population as well as among patients with Ca levels <1.0 mg/dL according to the upper limit of normal, P level was determined to be an independent factor affecting the indication for surgical treatment [β: -1.96,p=0.038, odds ratio (OR): 0.14, 95% confidence interval (CI): 0.02-0.89 and β: -2.3, p=0.034, OR: 0.10, 95% CI: 0.12-0.84 respectively].

Conclusion

We found a strong correlation between moderate hypoP and the severity of the biochemical manifestations of PHPT. In asymptomatic PHPT patients, moderate hypoP was predictive of surgical indication, independent of age and level of hypercalcemia.

## Introduction

Primary hyperparathyroidism (PHPT) was first described in Europe and North America in 1925, and it was simultaneously discovered on both continents [1]. At that time, classical PHPT only referred to symptomatic disease; its clinical picture was characterized by brown bone tumors, bone cysts, fractures (especially vertebral fractures), nephrocalcinosis, renal failure, peptic ulcers, and pancreatitis attacks [1]. Since the introduction of routine serum calcium (Ca) level measurement in the 1970s, PHPT has become the third most common endocrine disease worldwide [2]. Subsequently, the concepts of asymptomatic PHPT (aPHPT) and normocalcemic PHPT (nPHPT) have also been described in the literature [3]. This common endocrine disease presents with a high serum Ca level, and the diagnosis is confirmed by a high or inappropriately normal parathormone (PTH) level and a high urinary Ca (UCa) level [3].

Currently, the vast majority of PHPT patients (80%) are asymptomatic; they do not have the skeletal and renal manifestations found in classical PHPT [4]. At the Fourth International Workshop on the Management of aPHPT, surgical intervention was recommended for patients with subclinical disease, skeletal or renal end-organ effects, or evidence of a risk of disease progression [4]. This recommendation encompassed patients with serum Ca >1 mg/dL above the upper limit of normal [4]. According to the guidelines, surgical intervention is also recommended if serum Ca increases by <1 mg/dl in patients with one of the following conditions: osteoporosis (T-score ≤-2.5) or fracture on vertebral imaging, estimated glomerular filtration rate (eGFR) <60 mL/min, severe hypercalciuria (>400 mg/day), renal calculi or nephrocalcinosis and age <50 years [4]. Approximately 25% of initially asymptomatic patients may develop end-organ effects within five years of diagnosis; the likelihood of this progression appears to be unrelated to the severity of hypercalcemia [5].

In patients with PHPT, 25-hydroxyvitamin D (25OHD), the storage form of vitamin D, is generally low, whereas activated vitamin D [1,25-dihydroxyvitamin D (1,25OHD)] is close to the upper limit of normal and sometimes elevated [6]. Some but not all studies have shown that PHPT is more severe in patients with 25OHD deficiency than those with normal 25OHD levels; such patients have higher serum Ca, 1,25OHD, and alkaline phosphatase (ALP) levels and decreased P levels [7]. The role of P assessment in the clinical management of PHPT is currently uncertain [7]. To date, P levels have not been taken into consideration in PHPT diagnosis and treatment recommendations [4]. Meanwhile, in PHPT, sodium phosphate co-transporter type 2a (NPcT2a) protein expression decreases in the proximal renal tubule, increasing PTH and renal clearance of P [8]. Furthermore, serum P is usually in the lower half of the normal range in PHPT cases [8]. However, only some PHPT patients have markedly decreased serum P levels [8].

Few studies have investigated the relationship between the laboratory and clinical symptoms of PHPT and hypophosphatemia (hypoP). Two recent studies have emphasized that the presence of hypoP in PHPT cases is biochemically and clinically very important [9,10]. These studies recommended simultaneous routine monitoring of serum P, PTH, and Ca levels in PHPT patients and noted that moderate hypoP should be a criterion for parathyroidectomy in asymptomatic PHPT patients aged over 50 years old with a mildly increased serum Ca level [9,10].

In this study, we aimed to evaluate the effect of hypoP in the clinical management of PHPT by examining clinical and laboratory results.

## Materials and methods

Study design

This was a retrospective study involving 164 patients diagnosed with PHPT in the tertiary endocrinology, diabetes, and metabolic diseases clinic between September 2020 and June 2023. The study was approved by the Ethics Committee of the University of Health Sciences, Bursa Faculty of Medicine, Bursa City Hospital, and was conducted in accordance with the principles of the Declaration of Helsinki.

Inclusion criteria

Male and female patients aged over 18 years with symptomatic or asymptomatic PHPT were included in the study.

Exclusion criteria

Patients with PHPT due to nPHPT, tertiary or secondary hyperparathyroidism, familial hypocalciuric hypercalcemia, and familial multiple endocrine neoplasms were excluded from the study.

Study design and work plan

The participants' medical history, demographic data, and laboratory results were obtained from their medical files. All PHPT diagnoses and differential diagnoses were made according to the current guidelines [4].

The PTH reference range was 15-65 ng/L, and the Ca reference range was 8.8-10.2 mg/dL. Severe PHPT and severe hypercalcemia were defined based on high serum Ca levels (Ca ≥11.2 mg/dL), while mild hypercalcemia was defined based on a serum Ca level <11.2 mg/dL [4]. A serum 25OHD level <20.0 µg/L was accepted as the cut-off point for deficiency [11].

HypoP was defined as a confirmed serum level below 2.5 mg/dl. Regarding P levels, a level of >2.5 mg/dl was classified as normal (Group 3), a level of 2-2.5 mg/dl was regarded as mild hypoP (Group 2), a level of 1-1.99 mg/dl was considered as moderate hypoP (Group 1), and a level of <1 mg/dl was categorized as severe hypoP [12]. None of the patients in the present study had severe hypoP.

The clinical diagnosis of osteoporosis is based on the presence of a fragility fracture or a T-score ≤-2.5 standard deviations (SDs) in any region based on bone mineral density (BMD), as measured using dual-energy X-ray absorptiometry (DXA) [13]. Renal involvement is considered positive if a patient has a history of kidney stones or a diagnosis of calculi or calcinosis is made based on an ultrasound [4].

Measurement of biochemical and hormonal parameters

Serum intact PTH levels were measured using an immunoassay method (Cobas c801, Roche Diagnostics Corporation, Indianapolis, IN). Serum 25OHD levels were measured using the high total electrochemiluminescence method (Cobas e801, Roche Diagnostics Corporation). Total serum Ca, P, and albumin levels were measured using calorimetric methods (Cobas e702, Roche Diagnostics Corporation). GFR was calculated by using the following Chronic Kidney Disease Epidemiology Collaboration (CKD-EPI) creatinine formula:

141×min(Serum Cr(sCr)/κ, 1)α×max(sCr/κ, 1)-1.209×0.993age×1.018×1.159

where sCr is measured as mg/dL, κ is 0.7 for women and 0.9 for men, α is -0.329 for women and -0.411 for men, min is the minimum of sCr/κ or 1, and max is the maximum of SCr/κ or 1.

Statistical analysis

Preliminary tests were conducted to determine the distribution of the variables. Normally distributed results of the Kolmogorov-Smirnov test were expressed as mean ±SD; results that were not normally distributed were expressed as median and interquartile range (IQR) of 25-75 percentiles. Continuous variables with non-normal and normal distributions were analyzed using the Mann-Whitney U test and the t-test for unpaired samples, respectively. Binary logistic regression analyses were applied to determine whether the correlated data were independent factors. The level of statistical significance was set at p<0.05. All analyses were performed using IBM® SPSS Statistics version 20 (IBM® Corp., Armonk, NY).

## Results

A total of 164 PHPT patients fulfilled the study criteria and were included in the study. The mean age of the participants was 57.6 ±11.1 years; the female (F)/male (M) distribution was 133/31. There were 102 patients [19 males (18.6%) and 83 females (81.4%)] with severe PHPT. Asymptomatic disease was found in 68/164 patients (41.5%). Meanwhile, 16 patients (9.8%) had osteoporosis and renal stones, and 80 patients (48.8%) had either osteoporosis or renal stones. The frequency of renal stones was 25.4% in women and 37.2% in men; however, it was not statistically significant (p=0.22). The clinical, demographic, and laboratory characteristics of the patients are shown in Table 1.

**Table 1:** Clinical, demographic, and laboratory characteristics of the participants *Since it does not show a normal distribution, median and interquartile range (IQR) of 25-75 percentiles are given BMI: body mass index; Cr: creatinine; GFR: glomerular filtration rate; Ca: calcium; P: phosphorus; PTH: parathyroid hormone; UCa: urinary calcium excretion; 25OHD: 25-hydroxy vitamin D; ALP: alkaline phosphatase; DEXA: dual-energy x-ray absorptiometry

Variables	Values
Age, years, mean ±SD	57.6 +11.1
Female/male	133/31
BMI, kg/m^2^, mean ±SD	28.9 ±4.0
Cr, mg/dL, mean ±SD	0.73 ±0.20
GFR, mL/min, mean ±SD	90.1 ±17.9
Ca, mg/dL, mean ±SD	11.6 ±0.8
P, mg/dL, median (IQR)	2.50 (IQR: 0.92)*
PTH, ng/L, median (IQR)	151.0 (IQR: 118.5)*
UCa, mg/day, mean ±SD	352.9 ±188.2
ALP, IU/L, mean ±SD	105.5 ±43.2
25OHD, µg/L, mean ±SD	16.3 ±7.9
DEXA	
Lumbar vertebrae 1–4, T-score, mean ±SD	-1.88 ±1.44
Lumbar vertebrae 1–4, gr/cm^2^, mean ±SD	1.004 ±0.183
Femur total, T-score, mean ±SD	-1.53 ±0.88
Femur total, gr/cm^2^, mean ±SD	0.835 ±0.141
Kidney stone	27.40%
Osteoporosis	
Femur total	24.30%
Lumbar vertebrae 1–4	41.50%

The P level was <2.5 mg/dl in 78 patients (47.5%). Meanwhile, 25 patients (15.2%) had moderate hypoP. In terms of gender, 22.6% of the male patients and 13.5% of the female patients had moderate hypoP.

Based on the P levels, the patients were classified into three groups. Group 1 had higher serum Ca, serum PTH, UCa, and ALP levels as well as lower BMI and 250HD than Group 3 (Table 2). No differences were found between Groups 1 and 3 in terms of BMD, frequency of osteoporosis, or frequency of kidney stones (Table 2).

**Table 2:** Clinical, demographic, and laboratory characteristics of the study groups according to P levels *Since it does not show a normal distribution, median and interquartile range (IQR) of 25-75 percentiles are given. ^a^Comparison of Groups 1 and 2. ^b^Comparison of Groups 2 and 3. ^c^Comparison of Groups 1 and 3 BMI: body mass index; Cr: creatinine; GFR: glomerular filtration rate; Ca: calcium; P: phosphorus; PTH: parathyroid hormone; UCa: urinary calcium excretion; 25OHD: 25-hydroxy vitamin D; ALP: alkaline phosphatase; DEXA: dual-energy X-ray absorptiometry; FT: femur total; L1-4: lumbar vertebrae 1-4

Variables	Group 1 (n=25)	P-value^a^	Group 2 (n=53)	P-value^b^	Group 3 (n=86)	P-value^c^
Age, years, mean ±SD	52.2 ±11.4	0.29	58.5 ±10.3	0.61	57.3 ±12.3	0.14
Female/male	18/7	0.39	42/11	0.43	73/13	0.11
BMI, kg/m^2^, mean ±SD	25.6 ±3.9	0.058	30.2 ±3.3	0.86	29.0 ±4.0	0.018
Cr, mg/dL, mean ±SD	0.72 ±0.34	0.011	0.73 ±0.14	0.18	0.67 ±0.18	<0.001
GFR, mL/min, mean ±SD	81.4 ±23.8	0.26	78.4 ±15.7	0.70	93.5 ±18.0	0.15
Ca, mg/dL, mean ±SD	12.9 ±1.0	<0.001	11.8 ±0.5	<0.001	11.1 ±0.3	<0.001
P, mg/dL, median (IQR)	1.65 (IQR: 0.38)*	<0.001	2.20 (IQR: 0.35)*	<0.001	2.80 (IQR: 0.35)*	<0.001
PTH, ng/L, median (IQR)	340.0 (IQR: 455.3)*	0.068	170.0 (IQR: 103.5)*	<0.001	124.0 (IQR: 84.0)*	<0.001
UCa, mg/day, mean ±SD	414.6 ±168.5	0.88	433.9 ±209.9	0.003	291.5 ±161.4	0.026
ALP, IU/L, mean ±SD	156.7 ±140.2	0.088	100.0 ±33.4	0.022	95.9 ±40.6	0.001
25OHD, µg/L, mean ±SD	13.8 ±7.3	0.42	15.4 ±8.1	0.002	18.2 ±7.8	0.001
DEXA						
L1–4, T-score, mean ±SD	-1.65 ±0.85	0.42	-1.96 ±1.11	0.23	-1.77 ±1.62	0.88
L1–4, gr/cm^2^, mean ±SD	0.893 ±0.194	0.98	0.999 ±0.139	0.20	1.0232 ±0.201	0.37
FT, T-score, mean ±SD	-1.85 ±1.38	0.49	-1.42 ±0.87	0.65	-1.53 ±0.98	0.80
FT, gr/cm^2^, mean ±SD	0.822 ±0.124	0.94	0.863 ±0.158	0.36	0.821 ±0.140	0.49
Kidney stone	33.3%	0.35	23.1%	0.45	29.1%	0.69
Osteoporosis						
L1–4	36.4%	0.34	48.8%	0.27	38.2%	0.88
Femur	13.0%	0.78	15.6%	0.052	32.4%	0.071

The differences between the median P levels of those with and without renal stones [2.85 (IQR: 0.70) and 2.20 (IQR: 0.72), respectively; p=0.59], those with GFR <60 mL/min and those with GFR >60 mL/min [2.40 (IQR: 0.88) and 2.40 (IQR: 0.80), respectively; p=0.13], and those with and without osteoporosis [2.20 (IQR: 0.72) and 2.20 (IQR: 0.72), respectively; p=0.63] were not statistically significant. Regarding other parameters with a surgical indication, there were statistically significant differences between the median P levels of those aged <50 years and those aged >50 years [2.30 (IQR: 0.78) and 2.50 (IQR: 0.90), respectively; p=0.006] and those with UCa levels <400 mg/day and those with UCa levels >400 mg/day [2.60 (IQR: 0.80) and 2.20 (IQR: 0.60), respectively; p=0.007]. There was a significant difference between the median P levels of those who did and did not have a surgical indication [2.85 (IQR: 0.95) and 2.40 (IQR: 0.80), respectively; p<0.001]. Furthermore, the surgical indication frequency was significantly higher in Group 1 (100%) than in Group 3 (83.4%, p=0.024).

The patients were further classified into those with severe hypercalcemia, as evidenced by Ca levels >1.0 mg/dL above the upper limit of normal, and those with mild hypercalcemia, as evidenced by Ca levels <1.0 mg/dL. The clinical, demographic, and laboratory characteristics of the 102 patients with severe hypercalcemia and the 62 patients with mild hypercalcemia are shown in Table 3.

**Table 3:** Clinical, demographic, and laboratory characteristics of the participants according to Ca levels *Since it does not show a normal distribution, median and interquartile range (IQR) of 25-75 percentiles are given BMI: body mass index; Cr: creatinine; GFR: glomerular filtration rate; Ca: calcium; P: phosphorus; PTH: parathyroid hormone; UCa: urinary calcium excretion; 25OHD: 25-hydroxyvitamin D; ALP: alkaline phosphatase; DEXA: dual-energy x-ray absorptiometry; FT: femur total; L1-4: lumbar vertebrae 1-4

Variables	Mild hypercalcemia (n=62)	Severe hypercalcemia (n=102)	P-value
Age, years, mean ±SD	59.1 ±10.8	56.4 ±11.4	0.39
Female/male	52/10	83/19	0.68
BMI, kg/m^2^, mean ±SD	29.1 ±4.3	29.7 ±3.9	0.98
Cr, mg/dL, mean ±SD	0.63 ±0.16	0.80 ±0.20	0.01
GFR, mL/min, mean ±SD	94.9 ±16.9	86.4 ±18.0	0.081
Ca, mg/dL, mean ±SD	11.0 ±0.19	12.0 ±0.80	<0.001
P, mg/dL, median (IQR)	2.80 (IQR: 0.50)*	2.30 (IQR: 0.60)*	<0.001
PTH, ng/L, median (IQR)	147.5 (IQR: 89.5)*	217.2 (IQR: 174.5)*	<0.001
UCa, mg/day, mean ±SD	321.2 ±200.6	377.2 ±178.2	0.009
ALP, IU/L, mean ±SD	93.9 ±41.1	126.8 ±96.4	0.015
25OHD, µg/L, mean ±SD	18.1 ±7.9	15.6 ±7.9	0.046
DEXA			
L1–4, T-score, mean ±SD	-1.85 ±1.64	-1.91 ±1.29	0.51
L1–4, Gr/cm^2^, mean ±SD	1.004 ±0.200	1.004 ±0.171	0.69
FT, T-score, mean ±SD	-1.51 ±1.11	-1.55 ±0.67	0.51
FT, Gr/cm^2^, mean ±SD	0.825 ±0.165	0.843 ±0.122	0.70
Kidney stone	24.1%	23.1%	0.48
Osteoporosis			
L1–4	28.0%	29.3%	0.45
Femur	40.85%	42.4%	0.86

P levels were lower in the patients with severe hypercalcemia than in those with mild hypercalcemia [2.30 (IQR: 0.60) and 2.80 (IQR: 0.50), respectively; p<0.001]. Moreover, the low 25OHD levels in the patients with severe hypercalcemia could be considered a remarkable finding (Table 3). No differences in BMD, frequency of osteoporosis, or frequency of kidney stones were observed between the patients with severe and mild hypercalcemia (Table 3).

Among the patients with mild hypercalcemia, the differences in P levels between those with and without renal stones [2.90 (IQR: 0.75) and 2.80 (IQR: 0.50), respectively; p=0.59], those with GFR <60 mL/min and those with GFR >60 mL/min [2.60 (IQR: 0.80) and 2.80 (IQR: 0.50), respectively; p=0.56], and those with and without osteoporosis [2.85 (IQR: 0.52) and 2.75 (IQR: 0.52), respectively; p=0.94] were not statistically significant. Regarding other parameters with a surgical indication, the differences in P levels between those aged <50 years and those aged >50 years [2.65 (IQR: 0.50) and 2.85 (IQR: 0.52), respectively; p=0.011] and those with UCa levels <400 mg/day and those with UCa levels >400 mg/day [2.90 (IQR: 0.60) and 2.60 (IQR: 0.40), respectively; p=0.037] remained statistically significant in accordance with the whole sample population.

**Figure 1:**
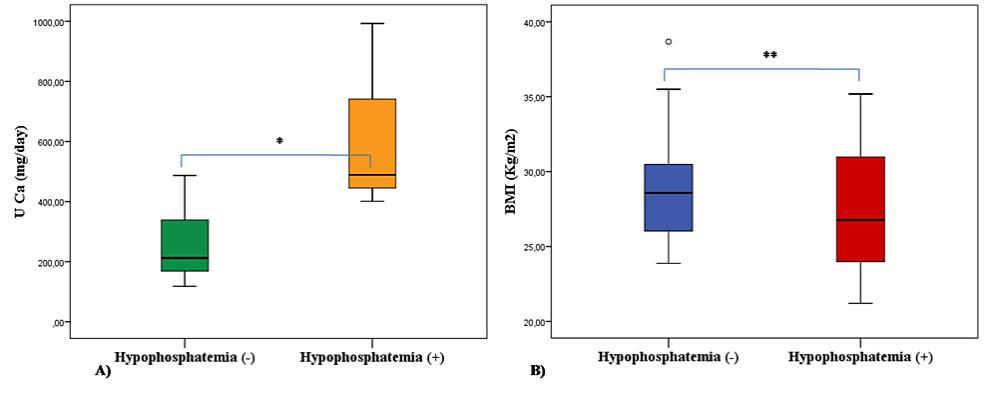
Comparison of UCa (A) and BMI (B) levels between patients with and without hypophosphatemia in the group with calcium levels <1.0 mg/dL compared to the upper limit of normal *p=0.013; **p=0.012 UCa: urinary calcium excretion; BMI: body mass index

As shown in Figure 1, among the patients with mild hypercalcemia, BMI was lower in those with hypoP than those without hypoP (24.0 ±6.1 and 29.0 ±3.8, respectively; p=0.012). In addition, UCa levels were higher in those with hypoP than those without hypoP (406.0 ±234.7 mg/day and 270.3 ±120.5, respectively; p=0.013).

Indications for surgical treatment

P was determined to be an independent factor affecting the indication for surgical treatment for the whole study population as well as among patients with Ca levels <1.0 mg/dL according to the upper limit of normal (Table 4). Of the 25 patients with moderate hypoP, nine (36%) were classified as aPHPT. Eight of these nine patients fulfilled the surgical indication criteria, i.e., serum Ca >1 mg/dl over the upper limit of normal and age <50 years; one had serum Ca <1 mg/dl. Furthermore, of the 25 patients with moderate hypoP, 24 (96%) met the serum Ca level surgical criterion. The patient who did not meet this criterion did meet the age criterion.

**Table 4:** Factors affecting indications for surgery based on binary logistic regression analysis *The whole study population. **In patients with calcium (Ca) levels <1.0 mg/dL compared to the upper limit of normal PTH: parathyroid hormone; P: phosphorus; OR: odds ratio; CI: confidence interval; OP indication: according to the guidelines, at least 1 indication for operation [4]

Dependent factor	Independent factors	B	Wald chi-square	p	OR	OR (95% CI)	
						Lower	Upper
Model 1 OP indication*	Constant	-8.29	1.26	0.26	0.000		
	Age	-0.43	2.10	0.58	1.65	0.29	9.60
	Ca	1.69	8.01	0.005	5.39	1.68	17.32
	PTH	0.001	0.10	0.76	1.01	0.99	1.04
	P	-1.96	4.32	0.038	0.14	0.02	0.89
	Gender	0.50	0.31	1.65	0.52	0.29	9.51
Model 2 OP indication**	Constant	7.47	0.75	0.39	0.000		
	Age	-0.61	3.13	0.077	0.94	0.88	1.01
	Ca	0.37	0.28	0.60	1.44	0.37	5.57
	PTH	-0.01	3.72	0.054	0.99	0.98	1.00
	P	-2.31	4.50	0.034	0.10	0.12	0.84
	Gender	1.23	1.28	0.26	3.44	0.41	29.09

## Discussion

The study findings revealed a significant association between moderate hypoP and severe PHPT. All patients with PHPT and moderate hypoP met at least one surgical indication criterion, as defined by the latest international guidelines. Furthermore, moderate hypoP was shown to be an independent factor in the surgical indication for PHPT.

Although every clinician considers serum P levels when assessing PHPT, the guidelines do not mention a cut-off value because serum P levels are normal in most PHPT patients [9,10]. In our study, serum P levels were normal in 64% of the patients. Furthermore, as noted above, P levels have not been included in PHPT diagnostic criteria or treatment indications [4]. Although serum P levels alone are considered unreliable for PHPT diagnosis, the serum Ca/P ratio has recently been proposed as an accurate index for PHPT diagnosis [14]. Of course, not all patients with PHPT have severe hypercalcemia, decreased GFR, kidney stones, osteoporosis, increased UCa, or decreased P levels. Our findings revealed a clear association between moderate hypoP and severe disease and surgical indication in PHPT cases.

HypoP has three basic mechanisms: increased renal excretion, decreased intestinal absorption, and shifts from extracellular to intracellular compartments. Renal hypoP can be divided into fibroblast growth factor 23 (FGF23)-mediated and non-FGF23-mediated causes [15]. P is freely filtered in the glomerulus and subsequently reabsorbed; however, proximal tubular reabsorption is inhibited by PTH and FGF23 [16]. In PHPT, which is the non-FGF23-mediated cause of hypoP, PTH strongly inhibits P reabsorption in the proximal tubule; a higher proportion of filtered P is excreted in the urine, leading to a decrease in the serum P level [17]. In PHPT cases, the 1,25OHD level is high and 25OHD is low due to hypoP because of the absence of physiological feedback response to calcium [6]. Meanwhile, in PHPT cases, intestinal P absorption is up-regulated by 1,25OHD [18]. It is rare for an individual to have PHPT and an FGF23-mediated disorder [15]. Previous studies have shown that 25OHD levels are even lower in patients with hypoP with PHPT [9,10,19]. In our study, 25OHD levels were lower in mild and moderate hypoP patients than non-hypoP patients and associated with higher serum Ca levels, i.e., severe PHPT, which is in line with the findings of other studies [9,10,19,20].

In the last decade, the understanding of phosphate metabolism has greatly increased with the study of rare phosphate homeostasis disorders [21]. The classic and most common inherited renal phosphate wasting disorder is X-linked hypophosphatemia (XLH), which presents with typical signs of rickets and is accompanied by hypoP [21]. ALP is usually elevated in XLH, although hypoP is present in cases where XLH, nephrolithiasis, and nephrocalcinosis are absent [21]. Hereditary hypophosphatemic rickets with hypercalciuria (HHRH) is a rare autosomal recessive disease [22]. Individuals with HHRH carry heterozygous or homozygous loss-of-function mutations in NPcT2a [22]; these mutations are independent of FGF23 and experience a loss of P in their urine. Also, these patients have hypoP and rickets [22]. Unlike XLH patients, HHRH patients have high 1,25OHD levels, which result in hypercalciuria due to increased intestinal Ca absorption and decreased PTH-induced Ca absorption in the distal renal tubules. Approximately 50% of patients with HHRH develop kidney stones and/or nephrocalcinosis [22]. Heterozygous NPcT2a mutations are frequently associated with isolated hypercalciuria (IH), which increases the risk of kidney stones or nephrocalcinosis threefold in affected individuals compared to the general population [23]. Bone disease is usually absent in patients with heterozygous NPcT2a mutations [23]. It is unknown whether secondary hyperparathyroidism will develop in HHRH patients or whether this disease will lead to osteoporosis [22,23].

The PHPT mechanism resembles that of HHRH. Increased PTH decreases NPcT2a protein expression in the proximal renal tubule and increases renal clearance of P, which sometimes results in a marked decrease in the serum P level [8]. As with the heterozygous form of this disease, not all patients with PHPT develop bone disease or kidney stones. In addition, severe PHPT may not be accompanied by nephrolithiasis or osteoporosis. In our study, PHPT severity in terms of biochemical indicators (high serum Ca, UCa, and PTH levels) was worse in hypoP patients than a normal level; however, severe PHPT was not associated with renal stones. These findings are consistent with those of Castellano et al. [9] and Düger et al. [10]. In fact, Castellano et al. stated that a gender-dragging effect cannot be ruled out because renal stones are generally more frequent in males with PHPT [24]. In the present study, UCa excretion was significantly higher in patients with hypoP than those with normal serum P levels, and this was one of the hit points for surgical indication in asymptomatic patients. This finding is in line with those of Castellano et al. [9] and Düger et al. [10].

Chronic hypoP causes osteomalacia with elevated alkaline phosphatase [16]. In our study, osteoporosis frequency did not increase in patients with hypoP; however, ALP levels were higher than a normal level, which is in accordance with the literature [16]. This could indicate osteomalacia, which could accompany chronic hypoP and 25OHD deficiency. In the present study, osteoporosis was not predicted by the severity of hypercalcemia or hypoP. Düger et al. [10] found that lumbar BMD values were significantly lower in patients with hypoP than a normal level; this result does not match our findings or those of Castellano et al. [9]. However, they found significantly lower P levels in PHPT patients with prevalent densitometric impairment at the radial site than at the vertebral and femoral sites. Meanwhile, our study and several others in the literature found that chronic hypoP did not cause a decrease in BMD [25]. This finding could indicate the presence of resistance to the effect of PTH at the bone level. We could not draw a definitive conclusion based on the present study.

Regarding the aforementioned genetic disorders, in some patients with PHPT, the PTH balance is disrupted by a trigger initiated by hypoP at the very beginning, and thus autonomy can start. In other words, hypoP is the earliest precursor of PHPT [26]. However, even if hypoP is associated with increased UCa, it might not be responsible for any renal stones or osteoporosis that might develop. Even severe hypoP might not cause renal stones, as in the aforementioned genetic diseases. Moreover, in genetic diseases associated with osteomalacia, it is unclear whether hypoP leads to osteoporosis in the chronic process.

In fact, decreased phosphate levels are a natural consequence of the PHPT mechanism. Our findings confirm the generally accepted inverse relationship between PTH and serum P levels [27]. HypoP associated with PHPT is usually mild; increased urinary phosphate excretion is offset by phosphate mobilization and increased intestinal absorption [28]. Serum P concentrations are rarely lower than 2.0 mg/dL (0.6 mmol/L). Our study endorses this finding because 48% of the patients had hypoP, while only 15% had a P level lower than 2 mg/dL. If P intake is low or the patient is taking phosphate-binding antacids at the same time, P might decrease further and fall below 1 mg/dL; however, none of the patients in our study had a value P level below 1 mg/dL. Similarly, in Castellano et al.’s [9] study, 41.9% of the participants had hypophosphatemia, 15% had moderate hypoP, and none had severe hypoP. Düger et al. [10] identified hypoP in 47% of their study participants, which included 14% with moderate hypoP; none had severe hypoP. Less than half of the participants in our study and the studies by Castellano et al. [9] and Düger et al. [10] had hypoP in the setting of normal serum P, suggesting that other factors kept serum P normal despite increased renal tubular loss [27].

HypoP is often overlooked due to its non-specific signs and symptoms. Diffuse muscle weakness is the most common symptom of hypoP, and weakness and fatigue are common symptoms of acquired hypoP [29]. In our study, BMI was significantly lower in patients with hypoP compared to those with normal P levels as well as patients with moderate hypoP compared to those with mild hypoP; a similar result was not obtained in any of the previous studies. There was no difference in BMI between the groups with severe and mild hypercalcemia. Among the patients with mild hypercalcemia, BMI was lower in those with hypoP than those without hypoP. However, we could not find any association between BMI and hypercalcemia; this might have been due to hypoP-induced indulgence.

This study has a few limitations, primarily its retrospective design. In addition, data related to urine P levels or dietary variations of the patients were not collected. Furthermore, we could not evaluate the radial BMD of the patients due to a lack of radial BMD data; 80% of the study population was female and recruited from a single center, which may limit the generalizability of the findings.

Moderate hypoP was strongly associated with severe PHPT, like severe hypercalcemia. All patients with severe PHPT had hypoP. However, just as osteoporosis and nephrolithiasis can develop independently of the degree of hypercalcemia, patients with PHPT, regardless of the degree of hypoP, may have different dynamics in this respect. Even concomitant increased UCa was not associated with nephrolithiasis in all patients, clearly indicating that PHPT complications are accompanied by different components on a patient-by-patient basis.

## Conclusions

Our findings have demonstrated that concomitant moderate hypoP is an independent parameter that reflects the severity of PHPT. Moderate hypoP could be a new criterion for PHPT management as well as an indication for surgery in patients with aPHPT who are over 50 years of age and have mild serum Ca elevation.
